# Individual Differences in Student Learning: A Comparison Between the Student Approaches to Learning and Concept-Building Frameworks

**DOI:** 10.3390/bs15081055

**Published:** 2025-08-04

**Authors:** Mark A. McDaniel, Christopher M. Wally, Regina F. Frey, Hayley K. Bates

**Affiliations:** 1 Center for Integrative Research on Cognition, Learning, and Education (CIRCLE), Washington University in St. Louis, St. Louis, MO 63130, USA; cwally@wustl.edu (C.M.W.); bates.h@wustl.edu (H.K.B.); 2 Department of Chemistry, University of Utah, Salt Lake City, UT 84112, USA; gina.frey@utah.edu

**Keywords:** student approaches to learning, concept-building, function learning, deep approach, surface approach, abstraction learner, exemplar learner, Modified Approaches and Study Skills Inventory (M-ASSIST), Revised Study Process Questionnaire (R-SPQ-2F)

## Abstract

In cognitive science and education research, learning has been described to occur at surface and deep levels. Learners are thought to orient more toward one of these approaches to learning versus the other. In cognitive science, this has been assessed with a concept-building framework using objective function learning tasks to classify students as exemplar (surface) or abstraction (deep) learners. In education, the student approach to learning (SAL) framework has used self-report survey measures to classify learners as relying on surface approaches or deep approaches to learning. In two studies, we directly compared these two frameworks using self-report data from the Modified Approaches and Study Skills Inventory (M-ASSIST) and the Revised Study Process Questionnaire (R-SPQ-2F) along with objectively determined concept-building classifications from a computer-based function learning task. Potential links between exemplar learning and surface approaches and between abstraction learning and deep approaches were not found. We discuss possible explanations for the absence of empirical links, including inaccuracies in students’ metacognitions regarding their learning, the measures, and possible differences between learning-content-dependencies of the survey responses versus content neutrality of the concept-building task. We conclude by suggesting directions for future work in assessing and comparing surface and deep learning across frameworks.

## 1. Background

A central idea that spans theory and research in education and cognitive science is that learning occurs at multiple levels. One common distinction is between learning at a surface level versus learning at a deep level. In general, a surface level approach involves memorization of provided examples, whereas a deep level approach pushes for understanding of underlying concepts. This distinction can be seen in education within the widely used student approaches to learning (SAL) framework (Biggs, 1993; Entwistle & McCune, 2004). In cognitive science, levels of understanding can be described as varying from focusing on surface, literal features to deep understanding involving formation of mental models (Johnson-Laird, 1983; Kintsch, 1988; Mayer & Gallini, 1990). Similarly, in terms of conceptual representations, such representations can rely solely on memory of the training examples or on abstractions generated to capture the relations of the training examples (e.g., Bourne, 1974; Koh & Meyer, 1991; D. R. Little et al., 2011; Medin & Schaffer, 1978; Nosofsky & Kruschke, 1992). Extending from this distinction is the idea that learners differ in their approaches to learning, with some learners adopting a relatively surface approach and others adopting a deeper approach. In this article, we review two frameworks that have attempted to establish and more completely characterize this posited individual difference in learners. The two frameworks have emerged in different spheres, one in the cognitive science domain and the other in the education domain, and with virtually no connections between the two made in the research literature. Yet, these frameworks share apparent similarities, prompting the question of whether they converge on the same individual difference, or at least have significant overlap. After describing the two frameworks, we report several novel studies to address this question.

### 1.1. Individual Differences in Learning Examples Versus Abstractions

Basic theoretical and empirical work from cognitive science has established that learning and understanding concepts can involve two fundamentally different representations. One kind of representation is based on learning the individual training examples (exemplar approach); the learned examples are then referenced when encountering new examples to categorize and draw inferences about the new examples (or problems) (e.g., Medin & Schaffer, 1978; Nosofsky & Kruschke, 1992). For instance, when learning about Lewis structure problems in general chemistry, a student could focus on memorizing the presented examples and solutions. These learned example problems and solutions are then relied upon to solve subsequent Lewis structure problems (e.g., when presented on a test). An example in biology could be when students learn about the concept of form follows function (which refers to the idea that the structure of a biological component (form) is adapted to perform a specific function). One could memorize different shape–function pairings of biological components and functions such as red blood cells providing oxygen or nerve cells (neurons) transmitting electrical–chemical signals. These relations could then be reproduced on a test.

Another kind of representation is based on an abstraction(s) from the trained examples that captures the critical features or basic principles that underlie the examples (Bourne, 1974; Koh & Meyer, 1991; D. R. Little et al., 2011). In this case, when learning about Lewis structure problems, a student would extract an understanding of the features and principles that underlie the particular solutions from the presented examples (e.g., that all non-hydrogen atoms would like a full shell of electrons, which is an octet, and that they obtain this octet by sharing electrons with another atom, either by forming a bond or by having lone pairs). These underlying principles are then applied to solve new Lewis structure problems (Frey et al., 2020). For the biology example, the student would use the illustrative examples—such as red blood cells—to abstract the concept of ‘form follows function,’ explaining why particular structures exist as they do and how those structures facilitate the functions they perform. For example, the biconcave shape of the red blood cells enables them to efficiently transport oxygen through narrow capillaries.

The key assumption of this framework is that individual learners can differ in terms of which approach they adopt when confronted with a learning challenge. In a range of conceptual tasks—category learning (J. L. Little & McDaniel, 2015), function learning (McDaniel et al., 2014), multiple-cue prediction learning (Hoffmann et al., 2014; Juslin et al., 2003), and skill learning (Bourne et al., 2010)—recent evidence supports this major tenet that an individual learner relies predominantly on either an exemplar or an abstraction approach to learn a particular conceptual task. Further, in a laboratory study, learners completed function-learning tasks and unrelated categorization tasks over the course of several weeks (McDaniel et al., 2014). Based on their extrapolation performance in the function-learning task, learners were identified as having oriented toward memorizing the particular training pairs (each input value/output value pair) or as attempting to abstract the underlying functional relations between inputs and outputs (a bilinear “V-shape”). Importantly, learners’ approaches on the function-learning task significantly predicted learners’ performances on subsequent category-learning tasks, such that those who displayed exemplar learning on the function-learning task showed example-driven behavior (on categorization transfer tests) consistent with an exemplar representation, whereas those learners who displayed abstraction concept-building also showed abstraction-driven categorization behavior. Thus, individual differences in concept-building tendencies tended to persist across very different learning tasks, even with those tasks presented several weeks apart. Converging with these findings, Herzog and Goldwater (in press) found that participants consistently displayed either an exemplar-learning approach or an abstraction approach across five distinct laboratory tasks (drawn from function learning, category learning, associative learning, and analogy).

Most critically for the present project, and for science learning in general, we have recently shown in several studies that the individual differences in concept-building tendencies, as indexed by the laboratory learning task from McDaniel et al. (2014), extend to the learning challenges in complex STEM topics (Frey et al., 2017, 2020; McDaniel et al., 2022, 2018). Students in chemistry classes and biology classes who displayed exemplar concept-building tendencies on the function-learning task (exemplar learners) tended to focus on learning particular example problems and solutions that are presented in class and homework (e.g., for Lewis structure problems, as mentioned above (Frey et al., 2020)). By contrast, students who abstracted in the function-learning task (abstraction learners) attempted to abstract the principles and concepts that underlie the class and homework problems (e.g., for Lewis structure problems). In terms of exam performances, the exemplar learners and abstraction learners, as identified on the basic function-learning task, performed equally well on course-exam problems that were similar to those presented in class (reproduction questions), but the exemplar learners performed poorly relative to the abstraction learners on exam items that were not similar to class problems, i.e., those that required generalization of the topic (transfer questions) (McDaniel et al., 2018, 2022). Critically, based on assessments of general ability, we were able to conclude that this divergence across students on transfer exam items did not simply reflect a difference in general ability or intelligence.

### 1.2. The Student Approaches to Learning Framework

A complementary framework often used in educational spheres is the student approaches to learning, SAL (Biggs, 1993; Biggs et al., 2001; Entwistle & McCune, 2004). The central idea is that some students adopt a deep approach to learning with the intention to understand the content, whereas other students adopt a surface learning approach with the intention to reproduce the content (Marton & Säljö, 1976). In this framework, this distinction emphasizes that students have different intentions when confronting a learning task (such as studying), and that these intentions in turn influence the strategies they use (see (Google et al., 2023), for applications of this framework to STEM learning). This categorical combination of intention and strategies (deep vs. surface) was termed “approaches to learning” (Marton & Säljö, 1997). For example, a key observation was that when students were given the same learning task (i.e., reading an academic article to prepare for answering questions about it), students varied in their descriptions of how they went about accomplishing the task (Marton & Säljö, 1997). The descriptions revealed a surface approach for some and a deep approach for others.

A student’s approach to learning is measured using a Likert-scale survey. These surveys were initially developed by a two-step methodology: qualitative analyses of students’ descriptions about their study processes followed by quantitative item creation. Many surveys were designed for generic learning contexts such as the Approaches and Study Skills Inventory for Students (ASSIST) (Tait et al., 1997), the Study Process Questionnaire (SPQ) (Biggs, 1987a, 1987b), and the Motivated Strategies for Learning Questionnaire (MSLQ) (Pintrich et al., 1991). Other surveys were developed from these original surveys, such as the revised two-factor Study Process Questionnaire (R-SPQ-2F) (Biggs et al., 2001), or modified for discipline-specific learning contexts, such as the Modified Approaches and Study Skills Inventory (M-ASSIST) in chemistry (Bunce et al., 2017; Malinakova, 2024). Findings on the relationship between learning approaches and student outcomes are mixed. Some studies show a positive correlation between a deep learning approach and student outcomes (e.g., Zeegers, 2001; May et al., 2012; Bunce et al., 2017), whereas others report no significant correlation between a deep-learning approach and outcomes (e.g., dos Santos Rodrigues & Gomes, 2020; Rowe, 2002; Maciejewski & Merchant, 2016).

### 1.3. The Present Studies

In considering the concept-building tendency and SAL frameworks that have been developed from different theoretical bases, research, and disciplines, a natural question is whether the two are converging on a similar idea to characterize individual differences in learning (cf. Goldwater et al., 2018, Experiment 4). Favoring this possibility is the theoretical premise that pushing for understanding (a deep approach in SAL) is a process that can involve abstracting the underlying principles or regularities of the target content (abstraction learners in the concept-building framework). By the same token, adopting a surface approach emphasizes reproduction of the target content (the SAL view); reproduction, at least in part, requires committing to memory the specific examples provided in the target content (exemplar learners in the concept-building framework).

To investigate the possible overlap between these two fundamental approaches concerning individual differences in learning, we conducted two related, novel studies. The studies adopted the same methodology of requiring a group of college students to complete both the self-report surveys used in the education literature based on the SAL framework and the function-learning task developed in the cognitive science learning literature. It warrants mention that the SAL instruments provide two indices for each respondent, a score reflecting their tendency toward a deep approach to learning and a score reflecting their tendency toward a surface learning approach; in contrast, the laboratory (function) learning task provides a categorical index indicating whether the respondent tends to use an abstraction approach or an exemplar approach to learning.

In these studies, we used correlational analyses and inferential statistics to test two competing hypotheses. Because the literature (to our knowledge) has not directly compared the SAL and concept-building frameworks, we did not have clear expectations for how they might be related. We thought it equally plausible that there may be positive associations between deep and abstraction learning and surface and exemplar learning, as described above, or that these approaches might not be related at all. Given this, we tested the following two hypotheses:

**Hypothesis 1.**  *Learners who adopt an abstraction approach to concept learning will report more extensive reliance on a deep approach to their learning than will learners who adopt an exemplar approach. On the other hand, learners who adopt an exemplar approach to concept learning will report more extensive reliance on a surface approach to their learning than will learners who adopt an abstraction approach.*

This hypothesis originates from the possible theoretical linkages that hinge on the idea that there is a close correspondence between learning processes and the consequent representations of what is learned. Surface learning processes (per SAL, committing items to memory) can be tied to literal representations that capture the items in the instructional material (e.g., for an instructional text, the representation would contain the words and sentences as presented in the text (Kintsch, 1988; Kintsch & van Dijk, 1978)). Similarly, when viewing a sample of values capturing a functional relation between two variables (e.g., amount of precipitation and crop growth), the representation would be those specific example values (DeLosh et al., 1997; McDaniel et al., 2014). Deep learning processes (per SAL, trying to gain understanding) are theoretically tied to representations that capture relations and abstractions among items. For instance, in an instructional text, it would be a mental model of what the text conveys (Kintsch, 1988; Mayer & Gallini, 1990); whereas, for presentation of function values, it would be an abstraction of the functional relationship conveyed by the training values (McDaniel et al., 2014).

Based on this hypothesis (and noting that our analyses coded an abstraction learner as “0” and an exemplar learner as “1”), there should be a significant *negative* correlation between concept-building tendency and the magnitude of endorsing a deep approach to learning, and there should be a significant *positive* correlation between concept-building tendency and the magnitude of endorsing a surface approach to learning. A related pattern that would solidify the merit of this hypothesis is that in a two-factor analysis of variance (ANOVA) with concept-building tendency and reported approaches to learning as the independent variables, there should be a significant cross-over interaction such that abstraction learners display a higher deep-approach to learning score than exemplar learners, whereas exemplar learners display a higher surface-approach to learning score than abstraction learners.

**Hypothesis 2.**  *The particular concept-building approach adopted by a learner has no discernible relationship to the self-reported approaches to learning endorsed by a learner.*

This possibility may be equally plausible and stems from important distinctions between the two frameworks. For example, SAL emphasizes a student’s *intention* in approaching a learning task (Marton & Säljö, 1976). Learners may not be able to achieve their intentions, however, especially in terms of trying to achieve understanding (e.g., Gouravajhala & McDaniel, 2025). For instance, in approaching a category learning task, some learners initially reported attempting to learn a rule (abstraction) that captured the range of training examples within a category (Gouravajhala et al., 2020). Despite their intention to learn a rule, by the end of training, a subset of these learners displayed an exemplar representation; i.e., their learned representations (for the categories) did not support a rule-based pattern of classifying novel category examples. The concept-building framework emphasizes, by contrast, the cognitive representations that learners actually acquire during learning—underlying abstractions (principles) that capture a concept or surface representations based on memorizing examples; it is these representations that ultimately reflect what the student is learning and index the degree to which individual learners achieve relatively deep learning.

Moreover, the SAL framework has relied on student self-reports of their learning approaches, thereby depending on the accuracy of students’ metacognitive awareness of their learning. Yet, the basic cognitive science literature has shown that students’ judgments of learning and of comprehension can be remarkably poor (Dunlosky & Metcalfe, 2008). Similarly, in a classroom chemistry study, a number of students judged that they had a good understanding of the principle even though think-aloud protocols revealed they did not (Frey et al., 2020). Therefore, poor metacognition—for instance when students self-report taking a deep approach to learning—may cloud the validity of classifications of learning approaches as deep or surface. For this reason or for the reason noted in the preceding paragraph (or both), there may be a negligible association between gauging students’ approaches to learning using common instruments inspired by SAL (introduced in the studies reported below) and the objective learning task inspired by the concept-building approach framework.

Repeatedly observing correlations that do not differ from “0” and finding no significant cross-over interaction in the ANOVAs (in each study) would be strongly consistent with this hypothesis.

## 2. Studies 1a and 1b

The SAL framework encompasses several complexities. One group of theorists assumes that students adapt their approaches to learning depending on the learning context, influenced by individual factors (e.g., student perceptions of the learning environment that are shaped by prior experience) and course-related factors (i.e., course design, the teaching and learning environment, and assessment procedures). For example, students are more likely to utilize surface approaches when faced with a learning environment that rewards rote memorization of facts. Further, the SAL framework has been developed in different discipline-specific learning contexts such as biology (dos Santos Rodrigues & Gomes, 2020; Nelson Laird et al., 2008; Lee et al., 2016), chemistry (Li et al., 2013; Sinapuelas & Stacy, 2015; Bunce et al., 2017; Atieh et al., 2020), mathematics (Maciejewski & Merchant, 2016; Lahdenperä et al., 2019), physics (Prosser et al., 2000; Selcuk, 2010), and engineering (Rowe, 2002; Zakariya et al., 2023). Consequently, the specific study strategies defined as deep and surface approaches to learning are assumed by some SAL proponents to vary across disciplines and learning environments (e.g., Sinapuelas & Stacy, 2015; Case & Marshall, 2004).

To capture the context dependency of the learning environment for determining in part a student’s approach to learning, Study 1a included an examination of the relationship between a student’s concept-building approach and their student-learning approach in a general chemistry classroom. Students in this class completed both the concept-building task and the Modified Approaches and Study Skills Inventory (M-ASSIST) survey, thereby enabling a direct classroom comparison of whether the deep and surface approaches in the SAL framework capture a similar characterization of individual learning differences based on the cognitive representations students construct during a learning task (measured by the concept-building task). The M-ASSIST is a shorter adaptation of the ASSIST survey^1^ developed specifically for chemistry and has been used in both general chemistry and organic chemistry (Bunce et al., 2017; Malinakova, 2022, 2024). The M-ASSIST consists of 12 items focusing on the deep approach (6 items) and the surface approach (6 items). The M-ASSIST was selected for the present studies because of its adaptation for chemistry courses and its focus on deep and surface approaches of the SAL framework.

Study 1b was motivated by an additional complexity in the SAL literature. Some theorists suggest that the two learning approaches—deep and surface—reflect overarching characteristics that transcend the particular learning context (Entwistle et al., 1979; Entwistle & McCune, 2004). With the deep approach, students are intrinsically motivated to understand the intentions of the educational tasks, to connect the task to their own real-world experiences, to integrate aspects of the tasks with prior knowledge, and to ask questions about the tasks. By contrast, with the surface approach, the students are driven by extrinsic factors, such as fear of failure, which lead to low-level cognitive activities like rote learning or selective memorizing without understanding. Additionally, they invest minimal effort to understand the task’s purpose, even when high-level cognitive engagement is required.

To the extent that the SAL deep and surface approaches to learning are overarching characteristics of the learner, then those characteristics might best be evidenced outside of a particular classroom context. To test this possibility, in Study 1b, we administered the M-ASSIST to students in a laboratory setting and compared these responses to their concept-building tendencies (determined in the same laboratory setting).

Hypothesis 1 (from the general introduction) would be strongly supported if at least one of these two studies (Study 1a or Study 1b) demonstrated significant associations between the M-ASSIST deep scale and a learners’ tendency toward an abstraction concept-building approach and between the M-ASSIST surface scale and learners’ tendency toward an exemplar-based concept-building approach. The absence of associations between the M-ASSIST survey responses and learners’ concept-building tendencies in both Studies 1a (reflecting the possibility of an assumption of course specific influences of students’ deep vs. surface approaches to learning) and 1b (reflecting an overarching tendency outside the context of a specific course) would support Hypothesis 2.

### 2.1. Method

#### 2.1.1. Participants

Study 1a examined students enrolled in a first-semester general chemistry course at a small health professions school in the midwestern United States in fall 2017. Note that the concept-building data for this study were partially reported in previous work (Frey et al., 2020), but the data related to the M-ASSIST was not analyzed and is reported here for the first time. The project was approved by the institutional review boards at the university where the data were collected (IRB ID no. 2017-39) and the university where the data were analyzed (IRB ID no. 201710100). Consent was provided by 108 out of 123 (87.8%) of these students. Study 1b included 77 students electing to participate in a research laboratory experiment as one of several options to fulfill a research credit requirement for their psychology courses at a mid-sized private research university in the midwestern United States in fall 2024. The project was approved by the university’s institutional review board (IRB ID no. 202308131).

#### 2.1.2. Concept-Building Task

Participants’ concept-building tendency (exemplar or abstraction learners) was determined using the same concept-building task established in previous work (McDaniel et al., 2014, 2018; Frey et al., 2017, 2020). Participants had no prior knowledge or exposure to the task, as it involved a fictional scenario with an alien organism and two non-existent elements. The task was completed on a computer, and participants were asked to imagine they had been hired by NASA to study a new organism found on Mars that absorbs an element called Zebon and releases an element called Beros. The goal of the task was to predict an output variable (quantity of Beros) based on the input variable (quantity of Zebon). Unknown to the participants, the input-output points followed either an inverted-V function (Study 1a) or a regular-V function (Study 1b).

**Figure 1:**
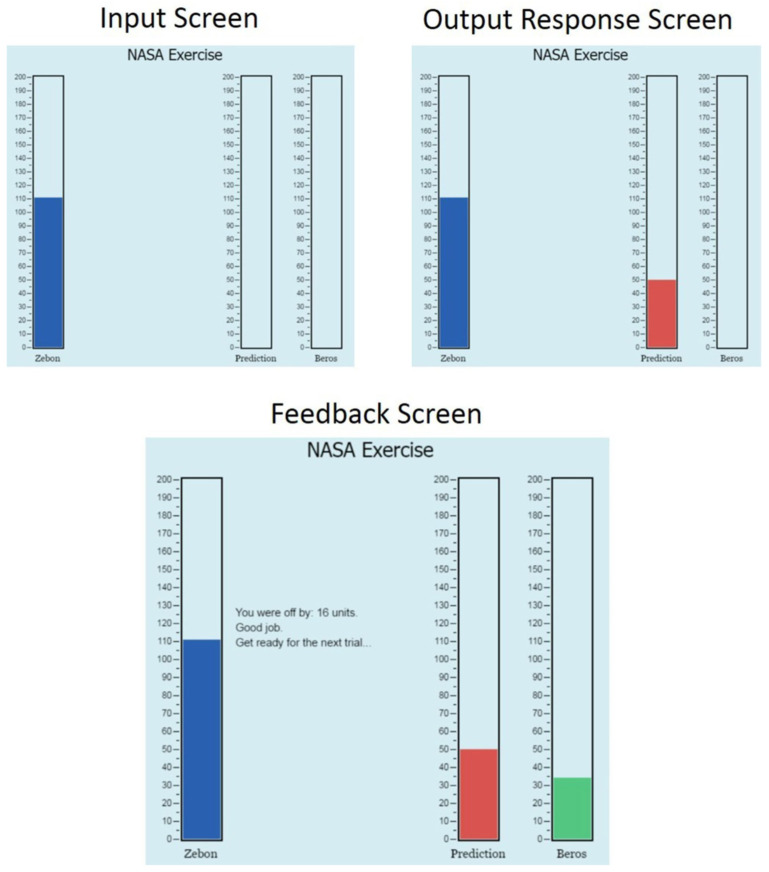
Screenshots from the concept-building task. ***Note.*** Adapted from (McDaniel et al., 2014). Copyright 2014 by the American Psychological Association.

Participants first completed a training phase, in which output predictions were made based on given input values, followed by feedback showing the correct value and the amount of error in their prediction. Figure 1 shows an example of a training trial with input, output, and feedback screenshots. In total, there were 20 unique input values (all odd numbers between 61 and 99 for Study 1a; all odd numbers between 81 and 119 for Study 1b), which were presented once each during a training block. Participants completed a total of 10 training blocks (200 individual trials), with the order of input values varying across each training block. No instructions were given as to how long to spend on each trial; the participants paced themselves throughout the task. Following each block, participants were shown their mean prediction error (mean absolute error, MAE) for that block. Additionally, after block two, participants saw their previous MAE and a message indicating whether they had reduced or failed to reduce their error (“Your accuracy IMPROVED. Keep up the good work!” vs. “Your accuracy DID NOT IMPROVE. Keep working to improve your predictions!”). After 10 training blocks, if the participant achieved a MAE <10, training concluded. In Study 1a, if participants had not met the threshold, up to three additional training blocks were presented, with training ending if MAE dropped below 10 on either block 11 or block 12. Training ended after block 13 (trial 260) for everyone in this version, regardless of whether the learning criterion was met. In Study 1b, training ended following block 10 for everyone.

Following the training blocks, participants completed a 36-trial transfer phase in which they predicted output values for novel (previously unseen) inputs. The procedure was similar except that no feedback was given after each trial. After making a prediction, participants saw the message “Prediction recorded. Get ready for the next trial.” The transfer phase featured 30 extrapolation trials, composed of odd numbers outside the training range (all odd numbers between 31 and 59 and between 101 and 129 for Study 1a; all odd numbers between 51 and 79 and between 121 and 149 for Study 1b); it also included six interpolation trials, featuring even numbers within the training range (64, 72, 80, 88, 94, and 100 for Study 1a; 84, 92, 100, 108, 114, and 120 for Study 1b). The extrapolation and interpolation trials were intermixed for the transfer phase. Participants took around 40 min on average to complete the full task.

**Figure 2:**
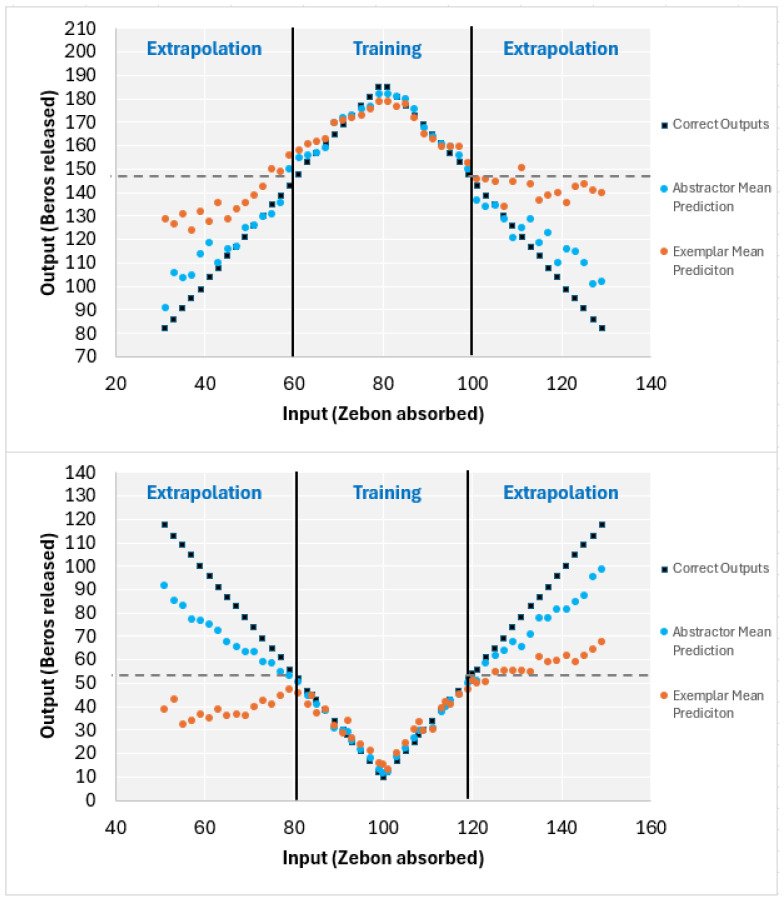
Input and output values from final training block and transfer trials for participants in Study 1a (**Top Chart**) and participants in Study 1b (**Bottom Chart**). ***Note*.** Vertical black lines represent the boundaries of the training range. Dashed horizontal lines represent theoretical exemplar model predictions based on learning the outputs at the boundaries of the training range and predicting those same outputs during extrapolation. Black squares represent the correct output values, based on the function. The blue circles represent the mean prediction across all abstraction learners for each input value. The orange circles represent the mean prediction across all exemplar learners for each input value.

As with the previous studies using the concept-building task, learners were classified using a two-step process. First, participants with a final training block MAE greater than or equal to 10 were classified as non-learners. The rest of the participants falling below the cutoff were considered “learners,” and were then further classified using their extrapolation MAEs from the transfer trials. By using a simple exemplar model as a comparison, one would predict a flat extrapolation extending from the edges of the training domain (represented by dashed horizontal lines in Figure 2). The functions used would result in a simple exemplar model making a prediction of 148 for every extrapolation trial in Study 1a and a prediction of 52 in Study 1b, leading to a MAE of 34.72 in both cases. Note that any set of predictions that average 148 or 52, respectively, and never overestimate the output value produce the same MAE of 34.72. The extrapolation MAE and surrounding 95% confidence interval were calculated for each learner.

If the upper-bound limit of the 95% confidence interval was below 34.72 (i.e., the learner’s predictions were statistically significantly better than a simple exemplar model), the learner was classified as an abstraction learner. This assumes that to outperform an exemplar model, learners must have extracted some information about the function rule during the training trials that was then applied during extrapolation. The remaining learners whose upper-bound extrapolation MAE values were greater than 34.72 (i.e., did not outperform a simple exemplar model) were classified as exemplar learners. The assumption is that these participants learned specific input-output associations but did not extract the function rule necessary to make accurate predictions on the novel transfer (extrapolation) inputs.

To represent these distinctions visually, Figure 2 displays the mean output predictions for each input value by abstraction and exemplar learners in Study 1a and Study 1b. Inspection of Figure 2 indicates that accuracies of predictions for abstraction and exemplar learners in the training range (interpolation) are both high (i.e., all learners look the same in the training range). In contrast, it can be seen that accuracy of extrapolation trials diverge, such that abstraction learners make predictions that follow the given function and exemplar learners do not extrapolate much beyond the value for the most extreme training point^2^ (i.e., tends to be relatively flat in predictions hovering around the value of the closest training point).

#### 2.1.3. The Modified Approaches and Study Skills Inventory (M-ASSIST)

The M-ASSIST is a 12-item measure developed by Bunce et al. (2017) to assess students’ approaches to studying (Bunce et al., 2017; see Supplementary Materials for full set of survey items). It is a brief, modified version of the Approaches and Study Skills Inventory for Students (ASSIST) (Tait et al., 1997). The M-ASSIST contains six items comprising a deep scale, reflecting deep approaches and strategies to learning, and six items comprising a surface scale, reflecting more superficial approaches and concerns about learning. Response options range from ‘disagree’ to ‘agree’ on a five-point Likert scale. Deep approach and surface approach scales were scored by averaging the individual item ratings across each scale. The measure was found to be a good fit for a two-factor structure across two semesters of data collected by Bunce et al. (2017) with model fit indices: CFI = 0.95, RMSEA = 0.05, and SRMR = 0.05 for the 1st semester, and CFI = 0.96, RMSEA = 0.04, and SRMR = 0.04 for the 2nd semester. Standardized factor loadings ranged from 0.36 to 0.70 for the deep items, and 0.43 to 0.82 for the surface items, with all *p*’s < 0.05.

#### 2.1.4. Procedure

For Study 1a, all students enrolled in the chemistry course completed the concept-building task following their first course exam during a supervised laboratory period approximately 5–6 weeks into the course. Students were given one-hour to complete the concept-building task. The M-ASSIST was completed 2–4 weeks later via an online survey at the student’s leisure during this time period. Only consenting students’ data were included in the analyses. Of the 108 consenting students, 54 (50%) were classified as abstraction learners, 28 (25.9%) exemplar learners, 25 (23.1%) non-learners, and 1 did not complete the task.

For Study 1b, participants were directed to a private room with a computer and proceeded to complete the concept-building task. Once the task was finished for participants, they were given five online surveys to complete, with the M-ASSIST included as the fourth survey. Participants were given up to 90 min to complete the concept-building task and surveys. Of the 77 consenting participants, 30 (39%) were classified as abstraction learners, 31 (40%) as exemplar learners, and 16 (21%) as non-learners.

#### 2.1.5. Analyses

All statistical analyses were conducted using SPSS version 29. To examine associations between deep approach and surface approach strategies on the M-ASSIST with abstraction and exemplar concept-building approaches, correlation coefficients were calculated. Concept-building tendency was dichotomously coded as 0 for abstraction learners and 1 for exemplar learners, and separate point-biserial correlations were computed with the deep approach and surface approach scores (treated as continuous variables). To increase sensitivity, we also included the continuous variable extrapolation mean absolute error (MAE) to index concept-building tendency and compared this with the deep and surface approach scores. A 2 (Concept-Building Approach: Abstractor vs. Exemplar; between-subjects) X 2 (M-ASSIST: Deep Approach vs. Surface Approach; within-subjects) mixed factor analysis of variance (ANOVA) was used to evaluate if abstraction learners endorsed deep approach items more strongly than surface approach items, and exemplar learners endorsed surface items more than deep items (an interaction effect). Statistical significance was set at *p* < 0.05 for all tests. As a general guideline for effect size estimates, Pearson’s *r* values of 0.1 are considered small, 0.3 medium, and 0.5 or higher large; partial-eta squared values (associated with the ANOVA effects) of 0.01 are considered small, 0.06 medium, and 0.14 large (Cohen, 1988).

Power analyses using G*Power 3.1.9.7 (Faul et al., 2007, 2009) were conducted to determine the power available to detect medium-size and large-size effects for Study 1a and Study 1b. At a significance level of *p* = 0.05, Study 1a (*N* = 82) provided 79% power to detect a medium correlation (99% power for large correlation) and 87% power to detect a medium effect size (99% power for large effect size) when testing the interaction between concept-building and learning approach using the M-ASSIST. Study 1b (*N* = 61) provided 66% power to detect a medium correlation (99% power for large correlation) and 73% power to detect a medium effect size (98% power for large effect) for the interaction. To provide greater power to detect associations between concept-building and student approaches to learning, data from Study 1a and Study 1b were pooled in a subsequent analysis. At a significance level of *p* = 0.05, this combined sample (*N* = 143) provided 96% power to detect a medium correlation and 98% power to detect a medium effect size.

### 2.2. Results and Discussion

Table 1 displays correlation coefficients for concept-building approach (as point biserial correlations), the continuous index used to determine learner category (extrapolation MAE), M-ASSIST deep approach scores, and M-ASSIST surface approach scores for Studies 1a and 1b. Concept-building approach was significantly positively correlated with the deep approach responses of the M-ASSIST in Study 1a, but not in Study 1b. The Study 1a correlation indicates that exemplar learners were more likely to endorse a greater number of deep approach items than abstraction learners—counter to Hypothesis 1. Concept-building approach was not correlated with the surface approach responses of the M-ASSIST in either study. Further, extrapolation MAE was not significantly related with deep or surface approach scores in either study. Finally, the deep and surface approach scales of the M-ASSIST were not significantly correlated with one another in either study.

**Figure 3:**
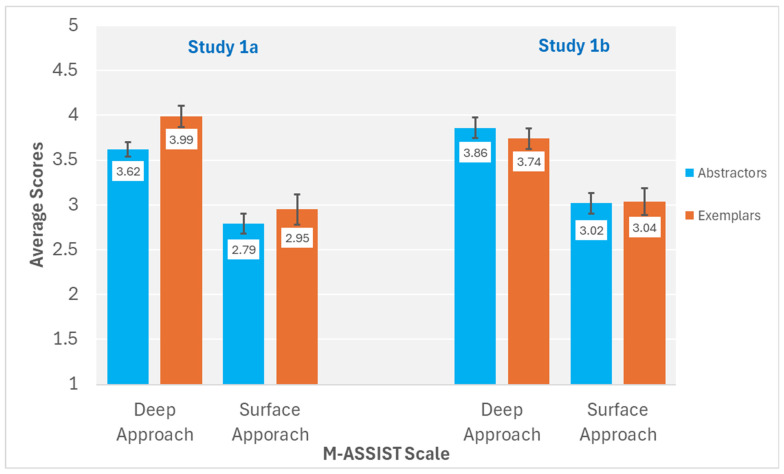
M-ASSIST deep approach and surface approach average scores for abstractor and exemplar learners in Studies 1a and 1b. ***Note*.** Error bars represent standard error of the mean. In Study 1a, there were 54 abstractors and 28 exemplars. In Study 1b, there were 30 abstractors and 31 exemplars.

Figure 3 displays the average scores for the deep approach and surface approach scales of the M-ASSIST for abstraction and exemplar learners in Studies 1a and 1b. For Study 1a, all learners endorsed significantly more deep approach items than surface approach items, *F*(1, 80) = 53.47, *p* <0.001, *η*^2^*_p_* = 0.401. Unique to Study 1a, exemplar learners endorsed items more strongly overall than abstraction learners, *F*(1, 80) = 5.56, *p* < 0.001, *η*^2^*_p_* = 0.065. Concept-building approach did not significantly interact with M-ASSIST learning approach, *F*(1, 80) = 0.68, *p* = 0.411, *η*^2^*_p_* = 0.008.

For Study 1b, as with Study 1a, all learners endorsed significantly more deep approach items than surface approach items, *F*(1, 59) = 33.88, *p* < 0.001, *η*^2^*_p_* = 0.365. However, in Study 1b exemplar learners and abstraction learners did not significantly differ in their endorsement of items, *F*(1, 59) = 0.20, *p* = 0.66, *η*^2^*_p_* = 0.003. Most importantly, concept-building tendency again did not significantly interact with M-ASSIST learning approach responses, *F*(1, 59) = 0.30, *p* = 0.585, *η*^2^*_p_* = 0.005.

#### Combined Analyses

Table 2 displays correlation coefficients for concept-building approach, extrapolation MAE, M-ASSIST deep approach, and M-ASSIST surface approach scores for the combined data of Studies 1a and 1b. Concept-building approach was not significantly related with the deep approach responses or with the surface approach responses of the M-ASSIST. Similarly, extrapolation MAE was not significantly correlated with deep or surface approach scores. Additionally, the deep and surface approach scales of the M-ASSIST were not significantly correlated with one another.

**Figure 4:**
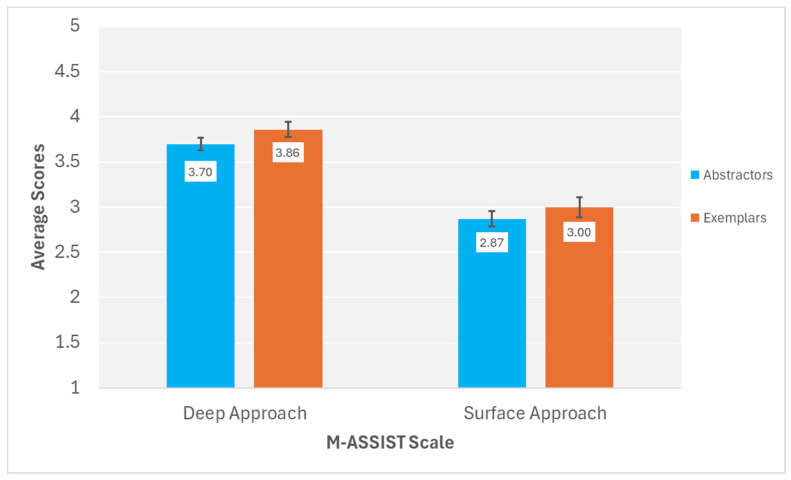
M-ASSIST deep approach and surface approach average scores for abstractor and exemplar learners for combined data of Studies 1a and 1b. ***Note*.** Error bars represent standard error of the mean. In the combined sample, there were 84 abstractors and 59 exemplars.

Figure 4 displays the average scores for the deep approach and surface approach scales of the M-ASSIST for abstraction and exemplar learners for the pooled data. Most importantly, converging with the individual analyses of Studies 1a and 1b, there was no hint of an interaction between concept-building tendency and self-reported learning approaches on the M-ASSIST, *F*(1, 141) = 0.02, *p* = 0.885, *η*^2^*_p_* = 0.000. Also as before, significantly more deep approach items were endorsed by learners compared to surface approach items, *F*(1, 141) = 87.05, *p* < 0.001, *η*^2^*_p_* = 0.382. With the pooled data, the tendency seen in Study 1a for exemplar learners to more strongly endorse M-ASSIST items regardless of approach (deep or surface) relative to abstraction learners was no longer significant, *F*(1, 141) = 2.96, *p* = 0.088, *η*^2^*_p_* = 0.021.

Hence, overall, when the results were considered across the general chemistry classroom and the laboratory settings, the patterns were straightforward. There were no significant correlations between the deep approach score from M-ASSIST and concept-building tendency or between the surface approach score from M-ASSIST and concept-building tendency. Reinforcing these correlation results, there was no crossover interaction in the ANOVAs between abstraction learning and deep approach scores or exemplar learning and surface approach scores.

Thus, these findings provide evidence for Hypothesis 2, which states that the concept-building tendency of a learner is not related to the learner’s self-reported approaches to learning. (Study 1a produced the anomalous finding that exemplar learners endorsed a greater number of deep approach items than abstraction learners, but this result did not stand in Study 1b or when combining the datasets. Perhaps, this was due, in part, to participants in Study 1a completing the concept-building task and M-ASSIST several weeks apart, during which some learners’ approaches to learning may have shifted. To avoid this possibility, Study 1b required participants to complete both assessments during the same session.) To provide further support for Hypothesis 2 of a non-relationship between these factors, a Bayesian alternative to traditional null-hypothesis significance testing was performed to test for an interaction (examined in the ANOVA) following the procedure outlined by Masson (2011) and originally formulated by Wagenmakers (2007). A Bayes factor value was computed (from the delta Bayesian Information Criterion) to determine the posterior probabilities of the null hypothesis model. Study 1a yielded a posterior probability of 0.865 for the null hypothesis, suggesting “positive” evidence for the null hypothesis model (i.e., no interaction) (following guidelines from (Raftery, 1995)). Study 1b yielded a posterior probability of 0.870 for the null hypothesis, again providing “positive” evidence for the null. The combined data yielded a posterior probability of 0.922 for the null hypothesis model, showing stronger positive evidence that concept-building tendencies were not related to students’ self-reports of deep or surface approaches to learning.

## 3. Study 2

Studies 1a and 1b relied on the M-ASSIST to determine students’ approaches to learning, which was designed specifically for use in chemistry courses. To better generalize outside of a chemistry learning context, in Study 2 we administered another commonly used SAL inventory, the two-factor Revised Study Process Questionnaire (R-SPQ-2F) to determine student approaches to learning (Biggs et al., 2001). In addition to being designed for generic learning contexts, the R-SPQ-2F has been used in mathematics (Maciejewski & Merchant, 2016), physics (Prosser et al., 2000), engineering (Zakariya et al., 2023), life sciences (Adkins et al., 2023; Leiva-Brondo et al., 2020), physiology (Thompson, 2024), and health sciences (LoGiudice et al., 2023). Additionally, the R-SPQ-2F provided a non-overlapping set of items with the M-ASSIST. It may be that the items on the R-SPQ-2F better reflect deep-understanding processes (deep approach to learning) that presumably lead to more abstract and encompassing cognitive representations and similarly better reflect surface learning processes (surface approach to learning) that support reliance on reproduction of the instructional examples (exemplar-based cognitive representations).

Again, in the present study, if Hypothesis 1 is supported, we should find significant associations between students’ self-reported approaches to learning and their concept-building tendencies (abstractor, exemplar). Alternatively, if Hypothesis 2 has merit, then the same pattern reported in Studies 1a and 1b should be observed; i.e., no evidence of an association between students’ self-reported approaches to learning and their objectively determined concept-building tendencies.

### 3.1. Method

#### 3.1.1. Participants

Study 2 was conducted in a research laboratory at a mid-sized private research university in the midwestern United States across two semesters (fall 2023 and fall 2024). Participants elected to participate as one of several options to fulfill a research credit requirement for their psychology courses. Consent was provided by 157 participants, of which 57 (36%) were abstraction learners, 56 (36%) were exemplar learners, 37 (24%) were non-learners, 5 (3%) did not complete the concept-building task, and 2 (1%) did not complete the R-SPQ-2F. The project was approved by the university’s institutional review board (IRB ID no. 202308131).

#### 3.1.2. The Revised Study Process Questionnaire (R-SPQ-2F)

The R-SPQ-2F is a 20-item questionnaire designed for use by educators to evaluate the learning approaches of their students (Biggs et al., 2001; see Supplementary Materials for full set of survey items). The scale has deep approach and surface approach scales, each with 10-items. Response options range from ‘never or only rarely true of me’ to ‘always or almost always true of me’ on a five-point Likert scale. The deep approach and surface approach scales were scored by creating a sum value of responses for each scale. The possible range of values for each scale is from 10 to 50. Biggs et al. (2001) examined the extent to which the items fit a deep approach and surface approach factor structure and yielded model fit indices of CFI = 0.99 and SRMR = 0.02 for their data, suggesting a good fit to the two-factor structure. Standardized factor loadings ranged from 0.31 to 0.63 for the deep items, and 0.38 to 0.67 for the surface items, with all *p*’s < 0.05.

#### 3.1.3. Procedure

The overall procedure and concept-building task were identical to that used in Study 1b; however, the order and number of surveys completed by participants varied by semester. In fall 2023, participants completed the R-SPQ-2F and a few short demographic questions; whereas in fall 2024, the R-SPQ-2F was completed last out of five surveys, the fourth being the M-ASSIST (the other surveys focused on motivational and mindset factors for purposes unrelated to this study). Participants were allowed up to 90 min to complete the concept-building task and surveys as part of one session.

#### 3.1.4. Analyses

The analysis plan was similar to that used in Study 1 with correlation coefficients and a mixed-factor ANOVA utilized to compare concept-building classification with learning approaches. The ANOVA was a 2(Concept-Building Approach: Abstractor vs. Exemplar) X 2(R-SPQ-2F: Deep Approach vs. Surface Approach). Power analyses were again conducted using G*Power 3.1.9.7 (Faul et al., 2007, 2009) to determine the power to detect medium and large effects. At a significance level of *p* = 0.05 with *N* = 113 (all abstraction and exemplar learners), the sample provided 90% power to detect a medium correlation and provided 91% power to detect a medium effect size when testing the interaction of concept-building and learning approach. There was 99% power to detect a large correlation and 99% power to detect a large effect size.

### 3.2. Results and Discussion

Table 3 displays correlation coefficients for concept-building approach (as point biserial correlations), extrapolation MAE (continuous index of concept-building tendency), R-SPQ-2F deep approach, and R-SPQ-2F surface approach scores. Concept-building approach was not related to the deep approach scale of the R-SPQ-2F or with the surface approach scale of the R-SPQ-2F. Extrapolation MAE was also not related to the deep or surface scales. The deep and surface approach scales of the R-SPQ-2F were significantly negatively correlated with one another.

**Figure 5:**
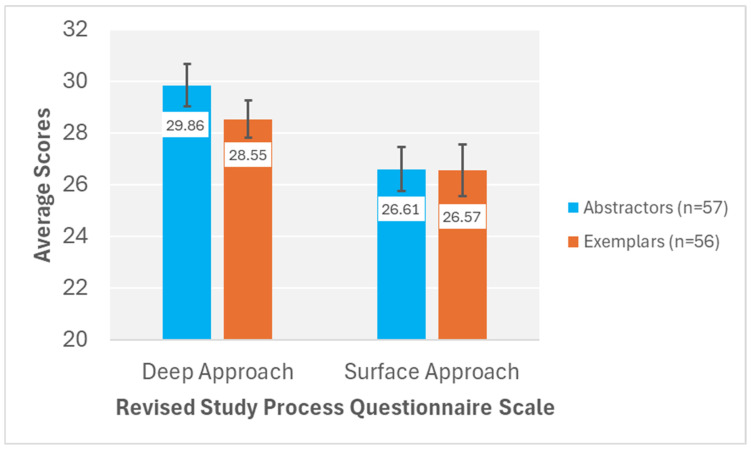
R-SPQ-2F deep approach and surface approach average scores for abstractor and exemplar learners. ***Note*.** Error bars represent standard error of the mean.

Figure 5 displays the average scores for the deep approach and surface approach scales of the R-SPQ-2F for abstraction and exemplar learners. Significantly more deep approach items were endorsed by all learners compared to surface approach items, *F*(1, 111) = 7.34, *p* = 0.008, *η*^2^*_p_* = 0.062. Exemplar and abstraction learners did not significantly differ in their endorsement of items on the R-SPQ-2F, *F*(1, 111) = 0.86, *p* = 0.356, *η*^2^*_p_* = 0.008. Most importantly, concept-building tendency did not significantly interact with R-SPQ-2F learning approach, *F*(1, 111) = 0.43, *p* = 0.514, *η*^2^*_p_* = 0.004.

### 3.3. Exploratory Analysis

Following Thompson’s (2024) comparison of the ASSIST and the R-SPQ-2F, as an exploratory analysis, we examined the relationship between the deep and surface approach items from the M-ASSIST and R-SPQ-2F using the fall 2024 subsample of participants, who completed both measures. Unlike the previous analyses, non-learners from the concept-building task were included in these correlations to achieve greater power, if they fully completed both the M-ASSIST and the R-SPQ-2F. Table 4 displays the correlation coefficients between the deep and surface approach scales from both the M-ASSIST and the R-SPQ-2F. The deep approach scales of the M-ASSIST and R-SPQ-2F were significantly highly correlated, and the surface approach scales of both instruments showed a significant moderate-to-strong correlation. There was also a small-to-moderate significant negative correlation between the surface scale of the M-ASSIST and the deep approach scale of the R-SPQ-2F, as well as a significant small-to-moderate negative correlation between the deep scale of the M-ASSIST with the surface scale of the R-SPQ-2F. The direction and strength of these relationships largely mirror that found by Thompson (2024), with the exception that Thompson did not find a significant negative relationship between the surface scale of the ASSIST with the deep scale of the R-SPQ-2F. Overall though, our analysis extends Thompson’s findings by using the more succinct M-ASSIST instead of the ASSIST and providing similar results with a different sample of participants.

Study 2 expanded on Study 1 by employing the widely used R-SPQ-2F as a measure of student approaches to learning and replicated the previous findings examining the relation of concept-building tendency with self-reported approaches to learning (indexed by the M-ASSIST) in a laboratory setting. There was no significant relationship between the deep approach score from R-SPQ-2F and the abstraction concept-building tendency or between the surface approach score from R-SPQ-2F and the exemplar concept-building tendency. These findings again lend support for Hypothesis 2, that the concept-building tendency of a learner is not related to the learner’s self-reported approaches to learning. To provide further evidence for Hypothesis 2, the Bayesian alternative to null hypothesis significance testing was again conducted as in Study 1. For the interaction in Study 2, the posterior probability for the null hypothesis being true was 0.895, providing “positive” evidence for the null hypothesis model (Raftery, 1995).

## 4. General Discussion

In this paper, we compared two frameworks used to measure students’ learning approaches: the concept-building framework and the student approach to learning (SAL) framework. We wanted to explore potential overlap between these frameworks in revealing how students learn. To investigate this issue, we conducted two related studies in which college students completed both SAL self-report surveys and a function-learning task designed to determine college students’ concept-building tendencies (abstraction vs. exemplar learning tendencies). For the SAL surveys, we used two common instruments in STEM education: M-ASSIST, an adaptation of ASSIST for chemistry, and R-SPQ-2F, which has been broadly used in many disciplines. These studies involved different student populations and were conducted in both a chemistry classroom and a research laboratory setting.

As developed in the introduction, it seemed plausible that these frameworks could be converging on a similar idea in individual differences among learners in the fundamental ways in which they approach learning challenges. However, across both studies, we found a consistent pattern: There was no detectable association between the deep versus surface learning approaches of the SAL framework (as measured by two SAL inventories) and the abstraction versus exemplar approaches from the concept-building framework. Specifically, in both studies (Studies 1a and 1b combined and Study 2), we found no significant correlation between a student’s concept-building approach and their SAL self-reports, whether using M-ASSIST or R-SPQ-2F. This lack of association remained consistent across both classroom and laboratory settings, despite the studies having sufficient power to detect medium size correlations. These findings suggest that the SAL framework instruments do not differentiate between exemplar and abstraction learners.

To further explore potential connections, we examined whether exemplar learners scored higher on surface approach items and abstraction learners scored higher on deep approach items. If the two frameworks measured a similar tendency, this pattern would be expected. However, across both SAL surveys, all students endorsed significantly more deep approach items than surface approach items, regardless of their concept-building approach. Thus, we found no empirical evidence linking these two theoretical frameworks. It could be that the lack of empirical overlap between the two frameworks stemmed from procedural, methodological, or participant-related factors affecting the behavior of the SAL inventories in our studies. Thompson (2024) found that in an undergraduate anatomy course, the R-SPQ-2F and ASSIST surveys aligned, with deep approach scores and surface approach scores having significant positive correlations across both instruments. This finding suggests that these two surveys are measuring similar constructs. If the M-ASSIST and R-SPQ-2F are functioning as expected in our studies, similar correlations should emerge. Our results confirmed this expectation. There were significant positive correlations between the M-ASSIST and R-SPQ-2F deep approach scores, as well as between their surface approach scores. Furthermore, we found significant negative correlations between deep and surface approaches across the two surveys. Hence, these findings support stability in our assessment of the deep and surface approaches, aligning with previous research. In the next section, we consider several alternative reasons why there was no support for an empirical overlap between the measurement indices that are commonly favored by these two frameworks.

### 4.1. No Empirical Overlap: Why?

One possible reason for no empirical overlap between the two frameworks is that the self-report inventories inspired by the SAL framework (Entwistle & McCune, 2004) may not provide a valid indicator of whether a particular student’s learning reflects a push toward understanding the content (deep approach) or reflects a push toward reproducing that content (surface approach). Students’ responses to some individual items could be inaccurate because they simply make careless responses or misinterpret the items. For instance, Mogashana et al. (2012) collected interviews from students who were instructed to elaborate on their responses to items on the Approaches to Learning and Studying Inventory (Entwistle & Ramsden, 1983) that target deep and surface approaches to learning. In some cases, in the interviews, students disagreed with their response(s), claiming that it was a mistake. In other cases, certain items confused students primarily because the entire statement was not well understood (e.g., “I just don’t get this one. It is a complicated statement”, p. 788), or because the students struggled in how to interpret a key word in the item (e.g., “trouble”). Additionally, interviews with undergraduates in an Anatomy class about their responses to the R-SPQ-2F survey found misalignments between their quantitative results and their qualitative data for at least 12 of the 20 items (Johnson et al., 2021). Students interpreted words in the items differently, and there were several compound items further complicating what exactly students were responding to.

In a related vein, these self-report inventories rely on the assumption that students’ metacognitions regarding their learning are accurate. Cognitive and education studies have shown; however, that learners’ metacognitions can be substantially inaccurate (Dunlosky & Metcalfe, 2008). With regard to the student approach to learning inventories, one challenge is that the student must be able to accurately report on how they learn. That is, students may believe that they are implementing a described activity, but in fact, they are not. These potential problems with the inventory content itself, along with possible metacognitive inaccuracies in how respondents report their approach to learning, may reduce the validity of the commonly used inventories for assessing student approaches to learning. As a result, this could weaken any empirical relationship between the SAL framework inventory assessments (e.g., the R-SPQ-2F) and the objective learning assessments used in the concept-building framework.

A second possible reason is that students’ responses to self-report inventories could in part reflect their intended approach, but not what they are able to achieve. Findings in classroom contexts (e.g., Frey et al., 2020; Wilujeng & Alvarez, 2025) consistently demonstrate that learners are not especially good at judging their level of understanding. Algebra students can express high confidence in incorrect answers, unaware that their understanding of the principles underlying the problem is flawed (e.g., Wilujeng & Alvarez, 2025). Similarly, Frey et al. (2020), using think-aloud protocols, confirmed that students’ incorrect answers on chemistry problems could be driven by incomplete understanding of the underlying concepts. Nevertheless, students were generally overconfident in the accuracy of these answers, suggesting that students were unaware that they had incomplete understanding of the requisite chemistry concepts. The point here is that some students may adopt a deep approach to learning—and thus endorse items on a SAL inventory that convey they are attempting to fit and relate ideas together, to understand the meaning of what they have to learn, to self -test until they have a complete understanding, and so on. Yet, because of poor meta-comprehension or because of having less skill at building organized cognitive structures (less-skilled structure builders (McDaniel et al., 2022)), these students do not achieve a deep understanding. Consequently, the association between responses on the SAL inventories and the objective learning patterns on the learning task used for identifying an individual’s concept-building tendency may not be as robust as underlying theory might suggest it should be. This may especially be the case for the deep-approach scale, where for a number of reasons just reviewed, the scale may not discriminate among the students who actually do achieve deep understanding versus those who attempt to understand the concept but ultimately do not.

A third potential reason for our finding of no empirical relation across the SAL and concept-building frameworks hinges on some SAL theorists’ emphasis that student approaches to learning are determined contextually (e.g., are dependent on specific course demands or content) (Sinapuelas & Stacy, 2015; Case & Marshall, 2004), which are revealed when students respond to the inventories within a particular course context. Because the concept-building task is neutral to course content and not completed with reference to particular course demands or content, concept-building approaches revealed by this task may not intersect with student approaches to learning revealed through SAL inventories administered within a course context (e.g., Study 1a). When SAL inventories are not administered within a course context (e.g., Study 1b, Study 2), it may be that they are not especially valid because they are not sensitive to the context dependencies that some SAL theorists believe are essential to understanding student approaches to learning.

In summary, our study found a disconnect between two well-known frameworks for understanding how students learn: the concept-building framework and the SAL framework. Despite theoretical overlap suggesting that deep learning approaches might align with abstraction-based concept-building and surface learning approaches might align with exemplar-based concept building, our empirical findings across two studies and multiple contexts did not support this assumption. The lack of correlation may reflect limitations inherent to self-report inventories, including inaccurate metacognitive judgments and item misinterpretation, as well as the potential for a misalignment between students’ intended and actual learning outcomes. Furthermore, contextual factors emphasized by SAL theorists may limit the utility of SAL inventories when used outside of specific course settings. These results suggest that while both frameworks offer valuable perspectives, they may be tapping into fundamentally different dimensions of student learning.

### 4.2. Study Limitations and Future Directions

The present study has some limitations worth acknowledging and also sets the stage for potential future directions into deepening our understanding of the relationship between these two frameworks, as well as how students learn. First, for classroom application, the concept-building task (function-learning) has some practical limitations. The task is relatively demanding, requiring about 45 min to complete. Moreover, to determine a student’s concept-building approach, a high level of learning in the training phase must be achieved (otherwise transfer patterns are indeterminate of individual differences in abstraction vs. exemplar learning). Unfortunately, 23% or more students typically do not meet this criterion in classroom studies (21–24% in the present studies conducted). It appears that part of this “failure” rate hinges on these students exerting less effort than those that meet the learning criterion (McDaniel et al., 2022). Incentives to increase motivation to perform well in the training phase might alleviate this problem. Alternatively, there is an existing category learning task that distinguishes between abstraction and exemplar learners but is less demanding and exhibits a much lower “failure” rate (J. L. Little & McDaniel, 2015). Future work could explore the extent to which this task provides an index of individual differences in concept-building approaches that is a valid indicator of classroom performance. Additionally, future work could explore having learners write down their problem-solving steps during the training phase. For example, we might see exemplar learners writing down input-output pairs for the training examples, whereas abstraction learners may attempt to graph the underlying function. Incorporating this aspect into the task could potentially reduce the number of non-learners or, at the very least, provide deeper insight into how different learners engage with the task.

A second limitation is that we did not examine the accuracy of students’ metacognitions regarding their responses to the approaches to learning inventories. Consequently, possible explanations that hinge on metacognition for why there was no empirical association between the SAL and concept-building frameworks remain speculative. Future work could examine the role of learners’ metacognition in influencing the accuracy of their self-reported approaches to learning. Following the issues raised in the preceding section related to students’ generally poor ability to estimate their own learning and judge their level of understanding, future studies might directly compare students’ SAL inventory responses with these students’ metacognitive accuracy. Two possibilities could be to (a) collect students’ inventory responses and sometime later conduct think-alouds for these students as they read a scientific text to evaluate the degree to which they are indeed activating surface versus deep learning approaches; or (b) administer comprehension questions to evaluate the degree to which deep understanding was achieved (e.g., Gouravajhala & McDaniel, 2025; McNamara et al., 1996) as a function of inventory response patterns.

A third limitation is that we did not assess course performance for our participants, to try to gauge the degree to which there are linkages between course performances (e.g., exam scores) and individual differences in learning approaches or in concept-building approaches. Future work could fruitfully examine the relationship between course achievement (exam questions or exam scores) and learners’ surface and deep approach tendencies on the one hand and their concept-building classification on the other hand. Existing research generated from each framework separately has done so, with somewhat divergent results. A deep learning approach should theoretically correlate positively with performance on transfer (or higher-order) questions. Yet, Thompson (2024), in an undergraduate human anatomy course, found no significant correlation between ASSIST or R-SPQ-2F learning approach scores (deep or surface) and exam scores, even when distinguishing between lower-order and higher-order questions. Similarly, LoGiudice et al. (2023), in two undergraduate health sciences courses, found no significant correlation between the R-SPQ-2F deep approach scores and student performance on transfer questions. By contrast, as reviewed in the Introduction, individual differences in abstraction learning tendencies versus exemplar learning tendencies are consistently associated with course achievement (Frey et al., 2017), particularly when course exams include transfer questions (McDaniel et al., 2018, 2022). However, the courses and course assessments across studies adopting the SAL inventories relative to those adopting the concept-building approach learning task have not been all that similar. Future studies that examine how both frameworks fare in predicting exam performances for the same students in the same courses could provide a substantial advance (with exam questions that target retention and questions that target transfer of course material).

## Figures and Tables

**Table 1:** Correlations for concept-building approach and M-ASSIST deep approach and surface approach scales in Study 1a (upper table section) and Study 1b (lower table section).

Study 1a Variable	1	2	3	4
1. Concept-Building Approach ^a^	-			
2. Extrapolation MAE	0.83 *	-		
3. M-ASSIST Deep Approach	0.29 *	0.19	-	
4. M-ASSIST Surface Approach	0.09	0.15	−0.08	-
Study 1b Variable	1	2	3	4
1. Concept-Building Approach ^a^	-			
2. Extrapolation MAE	0.87 *	-		
3. M-ASSIST Deep Approach	−0.10	−0.06	-	
4. M-ASSIST Surface Approach	0.01	0.04	−0.13	-

***Note*.**^a^ 0 = abstraction learners, 1 = exemplar learners. * *p* < 0.05. *n* = 82 for Study 1a correlations, *n* = 61 for Study 1b correlations.

**Table 2:** Correlations for concept-building approach and M-ASSIST deep approach and surface approach scales in combined Study 1a and 1b sample.

Variable	1	2	3	4
1. Concept-Building Approach ^a^	-			
2. Extrapolation MAE	0.83 *	-		
3. M-ASSIST Deep Approach	0.12	0.05	-	
4. M-ASSIST Surface Approach	0.08	0.09	−0.09	-

***Note*.**^a^ 0 = abstraction learners, 1 = exemplar learners. * *p* < 0.05. *n* = 143 for combined sample correlations.

**Table 3:** Correlations for concept-building approach and R-SPQ-2F deep approach and surface approach scales.

Variable	1	2	3	4
1. Concept-Building Approach ^a^	-			
2. Extrapolation MAE	0.88 *	-		
3. R-SPQ-2F Deep Approach	−0.11	−0.06	-	
4. R-SPQ-2F Surface Approach	−0.003	0.003	−0.28 *	-

***Note*.**^a^ 0 = abstraction learners, 1 = exemplar learners. * *p* < 0.05. *n* = 113 for all correlations.

**Table 4:** Correlations for M-ASSIST and R-SPQ-2F deep approach and surface approach scales.

Variable	1	2	3	4
1. M-ASSIST Deep Approach	-			
2. M-ASSIST Surface Approach	−0.083	-		
3. R-SPQ-2F Deep Approach	0.599 *	−0.294 *	-	
4. R-SPQ-2F Surface Approach	−0.378 *	0.400 *	−0.253 *	-

**Note.** * *p* < 0.05. *n* = 77 for all correlations; these include non-learners.

## Data Availability

The data presented in this study are available upon request from the authors.

## Notes

1 Many commonly used SAL surveys in STEM are based on the Approaches and Study Skills Inventory for Students (ASSIST) inventory (Tait et al., 1997). The ASSIST survey has 52 items that are related to approaches to studying and learning. Deep learning items ask about seeking meaning in the task, relating ideas, understanding how ideas fit together, interest in the ideas, and monitoring effectiveness; surface learning items focus on lack of purpose, unrelated memorizing, and fear of failure. This survey has been used in STEM courses such as engineering (Rowe, 2002), nursing (Trigwell & Prosser, 1991), and chemistry (Brown et al., 2015). The survey also features items comprising a strategic component, relating to study organization, time management, motivation, and assessment demands.

2 The observant reader may notice that there is a slight positive linear bias for exemplar learners seen in the left of the top chart and the right of the bottom chart of Figure 2, consistent with a ubiquitous bias in human function learning (see Busemeyer et al., 1997).

## Supplementary Materials

The following supporting information can be downloaded at: https://www.mdpi.com/article/10.3390/bs15081055/s1.
