# Supplementary material for: Individual Differences in Student Learning: A Comparison Between the Student Approaches to Learning and Concept-Building Frameworks
Source: Behav Sci (Basel). 2025 Aug 4;15(8):1055. doi: 10.3390/bs15081055 (PMC12383156; doi:10.3390/bs15081055)
Supplement: Supplementary file 1 [file behavsci-15-01055-s001.zip › behavsci-3703840-supplementary.pdf]

Supplementary Material

M-ASSIST Survey Items

Reprinted (adapted) with permission from Bunce, D. M., Komperda, R., Schroeder, M. J., Dillner, D. K., Lin, S., Teichert, M. A., & Hartman, J. R. (2017). Differential use of study approaches by students of different achievement levels. *Journal of Chemical Education*, *94*(10), 1415-1424. Copyright 2017 American Chemical Society.

Likert scale 1 to 5, with 1=disagree, 2=disagree somewhat, 3=unsure, 4=agree somewhat, 5=agree

Deep Scale Items

1. When I read an article or book, I try to find out for myself exactly what the author means.
2. When I'm working on a new topic, I try to see in my own mind how all the ideas fit together.
3. Ideas in course books or articles often set me off on long chains of thought of my own.
4. Before starting to work on an assignment or exam question, I think first how best to tackle it.
5. When I read, I examine the details carefully to see how they fit in with what's being said.
6. Before tackling a problem or assignment, I first try to work out what lies behind it.

Surface Scale Items

1. Often I feel I'm drowning in the sheer amount of material we're having to cope with.
2. Much of what I'm studying in this course makes little sense: it's like unrelated bits and pieces.
3. I often worry about whether I'll ever be able to cope with the work properly.
4. I concentrate on learning just those bits of information I have to know to pass.
5. I'm not really sure what's important in lectures so I try to get down all I can.
6. I often have trouble in making sense of the things I have to remember.

R-SPQ-2F Survey Items

Reprinted (adapted) with permission from Biggs, J., Kember, D., & Leung, D. Y. (2001). The revised two-factor study process questionnaire: R-SPQ-2F. *British Journal of Educational Psychology*, *71*(1), 133-149. Copyright 2001 John Wiley and Sons.

© Copyright 2000-2025 The British Psychological Society. The British Psychological Society is a charity registered in England and Wales, Registration Number: 229642 and a charity registered in Scotland, Registration Number: SC039452 - VAT Registration Number: 283 2609 94

Likert scale 1 to 5, with 1=this item is never or only rarely true of me, 2=this item is sometimes true of me, 3=this item is true of me about half the time, 4=this item is frequently true of me, 5=this item is always or almost true of me

1. I find that at times studying gives me a feeling of deep personal satisfaction.
2. I find that I have to do enough work on a topic so that I can form my own conclusions before I am satisfied.
3. My aim is to pass the course while doing as little work as possible.
4. I only study seriously what's given out in class or in the course outlines.
5. I feel that virtually any topic can be highly interesting once I get into it.
6. I find most new topics interesting and often spend extra time trying to obtain more information about them.
7. I do not find my course very interesting so I keep my work to the minimum.
8. I learn some things by rote, going over and over them until I know them by heart even if I do not understand them.
9. I find that studying academic topics can at times be as exciting as a good novel or movie.
10. I test myself on important topics until I understand them completely.
11. I find I can get by in most assessments by memorising key sections rather than trying to understand them.
12. I generally restrict my study to what is specifically set as I think it is unnecessary to do anything extra.
13. I work hard at my studies because I find the material interesting.
14. I spend a lot of my free time finding out more about interesting topics which have been discussed in different classes.
15. I find it is not helpful to study topics in depth. It confuses and wastes time, when all you need is a passing acquaintance with topics.
16. I believe that lecturers shouldn't expect students to spend significant amounts of time studying material everyone knows won't be examined.
17. I come to most classes with questions in mind that I want answering.
18. I make a point of looking at most of the suggested readings that go with the lectures.
19. I see no point in learning material which is not likely to be in the examination.
20. I find the best way to pass examinations is to try to remember answers to likely questions.

To obtain the main scale scores add items scores as follows:

Deep Approach = 1 + 2 + 5 + 6 + 9 + 10 + 13 + 14 + 17 + 18

Surface Approach = 3 + 4 + 7 + 8 + 11 + 12 + 15 + 16 + 19 + 20
